# Meningitis criptocócica asociada a hidrocefalia en un paciente inmunocompetente

**DOI:** 10.23938/ASSN.1067

**Published:** 2024-03-07

**Authors:** Julián Castro Castro, María Dolores Díaz López

**Affiliations:** 1 Servicio Gallego de Salud Complexo Hospitalario Universitario de Ourense Servicio de Neurología Ourense España; 2 Servicio Gallego de Salud Complexo Hospitalario Universitario de Ourense Servicio de Medicina Interna. Unidad de Enfermedades Infecciosas Ourense España

**Keywords:** Cryptococcus, Hidrocefalia, Inmunocompetencia, Meningitis, Ventriculostomía, Cryptococcus, Hydrocephalus, Immunocompetence, Meningitis, Ventriculostomy

## Abstract

La meningitis criptocócica es una infección infrecuente y con alta morbimortalidad, cuya presentación en pacientes inmunocompetentes es excepcional.

Presentamos el caso de un varón de 67 años que ingresó por un cuadro subagudo de alteración de la marcha e incontinencia urinaria. El examen neurológico reveló incapacidad para mantenerse en pie y deterioro de la memoria. Las pruebas de imagen craneales mostraron hidrocefalia tetraventricular obstructiva con áreas de gliosis en los pedúnculos cerebelosos. Se realizó tratamiento endoscópico de la hidrocefalia, con toma de muestras de líquido cefalorraquídeo en las que se observó crecimiento de *Cryptococcus neoformans*. El paciente mejoró con el tratamiento endoscópico y tras completar la terapia antifúngica intravenosa con anfotericina B liposomal y fluconazol durante diez semanas.

La meningitis criptocócica en pacientes inmunocompetentes se trata con antifúngicos. En raras ocasiones se presenta con hidrocefalia, situación que requiere tratamiento quirúrgico mediante derivaciones del líquido cefalorraquídeo o técnicas endoscópicas.

## Abstract (en)

Cryptococcal meningitis is an infrequent infection with high morbidity and mortality. Its presentation in immunocompetent patients is rare.

We present the case of a 67-year-old male who was admitted for subacute symptoms of gait disturbance and urinary incontinence. Neurological examination revealed inability to stand and memory impairment. Cranial imaging showed obstructive tetraventricular hydrocephalus with areas of gliosis in the cerebellar peduncles. Endoscopic treatment of hydrocephalus was performed and cerebrospinal fluid samples taken revealing the growth of *Cryptococcus neoformans*. The patient improved with the endoscopic treatment and after completing intravenous antifungal therapy with liposomal amphotericin B and fluconazole for ten weeks.

Antifungals are used to treat cryptococcal meningitis in immunocompetent patients. On rare occasions, it presents with hydrocephalus, a situation that requires surgical treatment using cerebrospinal fluid diversions or endoscopic techniques.

## INTRODUCCIÓN

La meningitis criptocócica es la infección fúngica oportunista más frecuente del sistema nervioso central en pacientes inmunodeprimidos; sin embargo, su presentación en pacientes inmunocompetentes es muy poco habitual y su diagnóstico suele ser más complejo^1^. Está provocada por *Cryptococcus neoformans*, que es un hongo heterobasidiomiceto encapsulado. La MC suele presentarse de manera subaguda o crónica, con cefalea intensa y menos habitualmente con signos meníngeos. En los pacientes inmunocompetentes la MC se asocia con mayor frecuencia con papiledema, hidrocefalia, déficit focal, crisis o criptococomas; con una presentación aguda en mayor porcentaje de casos^2^^,^^3^.

Presentamos el caso de un paciente inmunocompetente tratado en nuestro centro por un cuadro de alteración de la marcha, deterioro del nivel de consciencia e hidrocefalia obstructiva, cuya etiología fue una MC. Solo se han encontrado dos casos similares descritos en la literatura revisada.

## CASO CLÍNICO

Se presenta el caso de un hombre de 67 años que ingresó en nuestro centro por un cuadro de inestabilidad de la marcha con lateropulsión derecha e incontinencia urinaria de tres semanas de evolución. Como antecedentes a destacar, diabetes tipo 2 en tratamiento con metformina oral e hipertensión arterial; posible consumo excesivo de alcohol en el pasado. A la exploración en el momento del ingreso destacaba la imposibilidad para mantener bipedestación, con caída hacia atrás y hacia la derecha; así como dificultad para control postural en sedestación. La exploración del balance motor en extremidades y de los pares craneales era normal. A nivel cognitivo destacaba la desorientación temporal, la dificultad para el cálculo y la afectación de la memoria diferida.

**Figura 1:**
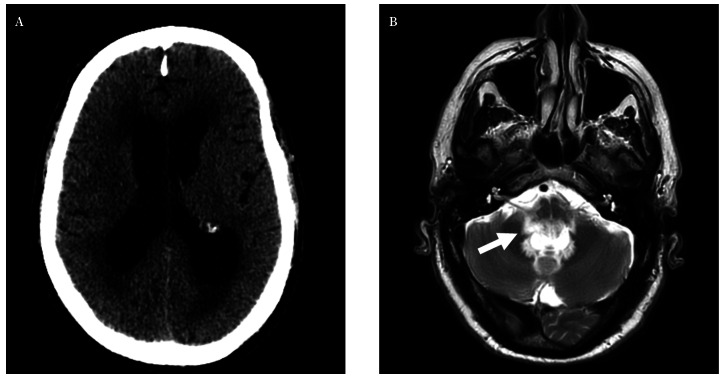
Pruebas de imagen realizadas al paciente. **A**. Tomografía computarizada craneal sin contraste realizada al ingreso, donde se aprecia dilatación ventricular con datos de trasudación transpendimaria. **B.** Resonancia magnética craneal en secuencia T2, con dilatación del IV ventrículo e hiperseñal alrededor de los forámenes de Luschka, observándose obstrucción a dicho nivel (flecha blanca).

**Figura 2:**
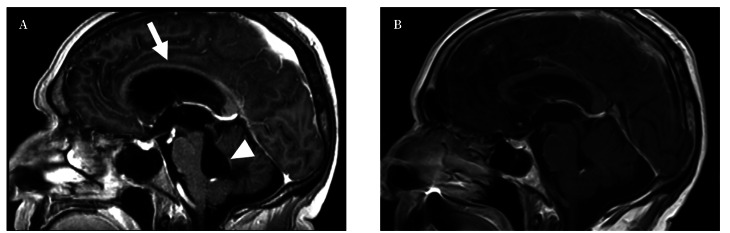
A. Resonancia magnética craneal en secuencia T1 con contraste, realizada a las 48 horas del ingreso. Se aprecia hidrocefalia con abombamiento superior y adelgazamiento del cuerpo calloso (flecha blanca) y dilatación del IV ventrículo (punta de flecha). B. Resonancia magnética craneal en secuencia T1 con contraste, realizada a las 2 semanas del tratamiento quirúrgico. Se observa la morfología normal del cuerpo calloso y del IV ventrículo, indicando la resolución de la hidrocefalia.

En la tomografía computarizada (TC) craneal realizada en urgencias (Fig. 1A) se apreciaba dilatación tetraventricular con trasudación. En el posterior estudio de resonancia magnética (RM) realizado a las 48 horas del ingreso se observaba esa misma hidrocefalia, sin lesiones que captaran contraste y con áreas de gliosis afectando a pedúnculos cerebelosos medio e inferiores y vermis (Figs. 1B, 2A).

Al ingreso se realizó el estudio preoperatorio habitual sin hallazgos a destacar ni en la radiografía de tórax ni en el estudio analítico con hemograma, bioquímica básica y coagulación.

Ante estos hallazgos de imagen se decidió tratamiento endoscópico de la hidrocefalia, que fue realizado a las 72 horas, con toma de muestra de líquido cefalorraquídeo (LCR) intraoperatoria. Se realizó fenestración endoscópica del suelo del tercer ventrículo. A destacar una presión de apertura de LCR de 22 mm Hg medida al realizar el procedimiento. El postoperatorio inmediato cursó sin incidencias. Los estudios analíticos y de microbiología de sangre periférica no mostraron datos a destacar. En el estudio de LCR se demostró pleocitosis con 335 células/µL (rango normal, RN: 0-5), de predominio monomorfonuclear (71%), con glucorraquia de 47 mg/dL (RN: 40-80, con glucemia simultánea de 96 mg/dL) y proteinorraquia de 189 mg/dL (RN: 15-60). En el cultivo de LCR se detectó crecimiento de *Cryptococcus neoformans.*

Este diagnóstico microbiológico se completó con el estudio de imagen mediante TC de tórax y abdomen, sin hallazgos de afectación en otras localizaciones. Se realizó despistaje de inmunosupresión, incluyendo serologías de VIH, VHB y VHC que fueron negativas, con valores de linfocitos 2.250/mm^3^ (RN: 1.300-4.000) y recuento de inmunoglobulinas IgG 1.000 mg/dL (RN: 650-1.600), IgM 200 mg/dL (RN: 54-300) e IgA 180 mg/dL (RN: 40-350). Los valores de hemoglobina glicosilada: 5% (RN: <5,7%) y de función hepática también fueron normales; albúmina: 5 g/dL (RN: 3,4-5,4), fosfatasa alcalina: 100 UI/L (RN: 44 a 147), transaminasa alcalina (ALT): 50 UI/L (RN:5-60), aspartato aminotransferasa (AST): 33 UI/L (RN: 10-34), gamma-glutamil transaminasa (GGT): 45 UI/L (RN: 5-80) y bilirrubina total: 1 mg/dL (RN: 0,1-1,2).

Ante los hallazgos de infección por *C. neoformans*, y al no disponer en nuestro centro de flucitosina, se instauró tratamiento intravenoso con anfotericina B liposomal (5 mg/kg/día) y fluconazol (800 mg/día) durante dos semanas. Pasado ese tiempo se realizó una punción lumbar para estudiar el LCR, observándose normalización de los parámetros bioquímicos y de la presión de apertura, y ausencia de *C. neoformans*. Se continuó tratamiento intravenoso con fluconazol oral (800 mg/día) durante ocho semanas más.

La evolución del paciente fue favorable, con recuperación neurológica progresiva. En la primera semana tras la cirugía mejoró la deambulación y la inestabilidad, mejorando el resto de síntomas neurológicos tras finalizar el tratamiento antifúngico. Al En la RM de control realizada a las dos semanas se comprobó la resolución de la hidrocefalia (Fig. 2B).

## DISCUSIÓN

Las infecciones criptocócicas del sistema nervioso central son infecciones oportunistas comunes en pacientes inmunodeprimidos, sobre todo en los trópicos (África subsahariana y el sudeste asiático). Suele afectar a pacientes con alteración de la inmunidad celular, como aquellos en estadios avanzados de infección por VIH, con diabetes, en tratamiento crónico con esteroides, trasplantados, o bajo tratamiento inmunosupresor^3^. Nuestro paciente era inmunocompetente, situación que, en los países occidentales, supone menos del 20% de los casos^1^. Los criptococos afectan habitualmente al tracto respiratorio causando una infección asintomática; posteriormente se diseminan de manera hematógena al cerebro^2^.

La meningitis criptocócica suele presentarse como una meningitis crónica o subaguda, siendo infrecuente con clínica aguda. El síntoma más característico es la cefalea intensa refractaria al tratamiento, presente en más del 75% de los pacientes; la fiebre solo se observa en el 65% de los casos. Alrededor del 20% de los pacientes pueden presentar alteración del nivel de alerta y menos del 8% sufren crisis comiciales. La rigidez de nuca solo está presente en el 30% de los casos. Como se mencionó previamente, la hidrocefalia y el edema de papila son manifestaciones clínicas más habituales en los pacientes inmunocompetentes^3^^-^^5^. La hidrocefalia puede deberse a la alteración de la absorción y la salida del LCR debidas a la acumulación de los polisacáridos del hongo en las granulaciones aracnoideas, o bien por la formación de adherencias en las vías normales de circulación del LCR^6^.

La prueba fundamental para diagnosticar la meningitis criptocócica es el análisis del LCR, que suele mostrar pleocitosis linfocitaria con proteínas elevadas y consumo de glucosa. El diagnóstico se realiza mediante aislamiento del hongo en cultivo de líquido cefalorraquídeo o identificación del antígeno criptocócico en análisis de sangre, como en el caso presentado^1^^,^^7^.

Las pruebas de neuroimagen más habituales, al igual que en el caso que presentamos, son la TC como prueba inicial para detectar la hidrocefalia o la RM con contraste que define mejor la existencia de lesiones ocupantes de espacio como los criptococomas^8^.

La terapia antifúngica habitual variará dependiendo del estado inmune del paciente, pero suele consistir en anfotericina B y 5-flucitosina intravenosas durante la fase de inducción por un periodo mínimo de dos semanas, seguida de una pauta de consolidación de 10-12 semanas de fluconazol oral, como se realizó en nuestro paciente. La fase de mantenimiento en pacientes inmunodeprimidos se realiza con fluconazol 200 mg/día por un periodo mínimo de un año^6^^,^^8^^,^^9^.

Se han descrito diversas opciones de manejo de la hidrocefalia asociada a la meningitis criptocócica. Lo más habitual es el uso de derivaciones externas de LCR como los drenajes ventriculares o los drenajes lumbares en caso de hidrocefalia comunicante. Algunos autores intentan el cierre progresivo del drenaje tras haber iniciado el tratamiento antifúngico. En caso de que el paciente no tolere el cierre del drenaje se procede a colocar una derivación ventriculoperitoneal de manera definitiva. En aquellos pacientes que presentan datos de hidrocefalia obstructiva existe la opción de la ventriculostomía endoscópica del tercer ventrículo como la que empleamos en nuestro paciente, evitando que sea portador de una derivación de por vida y además puede ser realizada aunque no haya negativizado los cultivos de LCR^10^^,^^11^.
